# Ferrous picolinate: a novel fortificant for sensitive dairy products

**DOI:** 10.3389/fnut.2026.1740721

**Published:** 2026-05-11

**Authors:** Edwin Habeych, Sylvie Merinat, Belén Sanchez-Bridge, Magalie Sabatier, Nicola Galaffu

**Affiliations:** 1 Nestlé Development Centre - Chilled Dairy, Beuvillers, France; 2 Nestlé Research, Vers-chez-les-Blanc, Lausanne, Switzerland; 3 Nestlé Institute of Food Safety and Analytical Sciences, Société des Produits Nestlé, Lausanne, Switzerland; 4 Nestlé Institute of Health Science, Societé des Produits Nestlé, Lausanne, Switzerland

**Keywords:** bioaccessbility, fortification, iron, picolinic acid, polyphenol-rich food, shelf-life, dairy, yoghurt

## Abstract

Ferrous picolinate is introduced as a novel iron fortificant designed to overcome the challenges of iron supplementation in polyphenol-rich dairy products. This study evaluates its stability and bioaccessibility in ambient milky desserts containing 25% fruit, stored under accelerated shelf-life conditions for 4 months. Colorimetric analysis demonstrated that ferrous picolinate does not alter the color of polyphenol-rich desserts, outperforming conventional fortificants. Furthermore, *in vitro* digestion coupled with Caco-2 cell model revealed iron bioaccessibility equivalent to ferrous sulfate, the current gold standard. These findings suggest that ferrous picolinate offers a promising solution for iron fortification in sensitive food matrices, combining high bioavailability with minimal sensory impact.

## 1 Introduction

Micronutrient malnutrition remains a global health concern, affecting billions and often manifesting as subclinical deficiencies (1). Iron Deficiency Anemia (IDA) is one of the biggest nutritional deficiencies, prevalent in both developing and industrialized countries and primary affecting women, infants and young children (2). In developing regions, IDA is primarily attributed to low dietary intake of bioavailable iron from monotonous plant-based diets.

Preventive strategies include dietary diversification, biofortification, and food fortification, with the latter recognized as the most effective approach for improving nutritional status without altering dietary habits (2). Despite its efficacy, iron fortification presents technical challenges, particularly in sensitive dairy matrices, hereafter defined in this manuscript as polyphenol-rich systems such as fruit-containing products, where iron–polyphenol complexation can result in off-color formation. Common iron compounds, such as ferrous sulfate, can induce undesirable sensory changes in fortified foods due to interactions with bioactive components, resulting in color changes, metallic taste, and rancidity (3, 4). Less soluble iron sources, like ferric pyrophosphate, offer improved stability but compromise bioavailability (5). To overcome these challenges, a range of innovative strategies has been developed:

- Absorption enhancers: the addition of compounds like ascorbate (vitamin C) can significantly boost iron absorption, while certain phosphates have also shown promise in improving bioavailability (6, 7).
- Chelated iron: utilizing chelated iron compounds—such as sodium ferric ethylenediaminetetraacetate (NaFeEDTA), ferrous bisglycinate, or multivalent metal salts like Ca^(2+)^–Fe^(2+)^ pyrophosphate—can enhance both stability and absorption (4, 8).
- Microencapsulation: encapsulating iron salts within protective matrices helps shield the iron from interactions with food components, thereby preserving sensory qualities and improving stability (9, 10).
- Biological systems: employing biological systems, such as iron-enriched yeast or the fungus *Aspergillus oryzae*, introduces highly bioavailable forms of iron into food products (11, 12).
- Particle size reduction: decreasing the particle size of poorly soluble iron compounds increases their surface area, which can enhance solubility and absorption (13).

Among these approaches, chelation and complexation of iron ions have shown the greatest promise for achieving high bioavailability while maintaining product stability (5).

Picolinic acid an isomer of vitamin B3, a naturally occurring chelator (14) and metabolic product of L-tryptophan, has been identified as a key facilitator of mineral absorption in mammals. Its presence in human milk is associated with enhanced zinc bioavailability (15), suggesting potential benefits for iron fortification. This proof-of-concept study investigates the stability and bioaccessibility of ferrous picolinate in fruit-containing dairy products, comparing its performance to commercial iron fortificants. Stability was assessed via color analysis as reported earlier (3), and iron uptake was evaluated by epithelial colorectal adenocarcinoma (Caco-2) cell model. These results complement previous human intervention trials and support the use of ferrous picolinate as a fortificant in sensitive food matrices (16).

## 2 Materials and methods

### 2.1 Materials

2-picolinic acid (C_6_H_5_N_2_; Sigma-Aldrich), ferrous sulfate (FeSO_4_ x 7H_2_O; Dr. Paul Lohmann) in short FeSO_4_, ferric pyrophosphate (Fe_4_(P_2_O_7_)3*xH_2_O; Dr. Paul Lohmann) in short FePP, Ferrazone® (C_10_H_12_O_8_N_2_FeNa*xH_2_O; AkzoNobel) in short NaFeEDTA, LipoFer™ (Encapsulated FePP from LipoFood), ammonium thiocyanate and ferrozine (Sigma Aldrich) were supplied from commercial sources and used without preliminary purification. Shelf-stable milky desserts with fruit containing: 25% fruit (15% banana; 10%: strawberry); calcium: 75 mg/100 g; magnesium: 15 mg/100 g; zinc: 0.75 mg/100 g, was supplied by the Nestlé Factory in Epinal (France).

### 2.2 Iron analysis

The iron content in ferrous picolinate was determined using a Vista MPX ICP-AES (Varian AG, Zug, Switzerland) equipped with a standard glass concentric nebulizer and a cyclone glass spray chamber. In short, approximately 200–400 mg of sample were mineralized in duplicate in a Mars Microwave digestion system (Model Mars 5, CEM, USA) using Xpress microwave bombs and 3 mL HNO_3_ s.b./2 mL H_2_O_2_ 30%. Sample aliquots were diluted to 15 mL after addition of scandium and caesium as internal standards (1 and 2,000 mg/L). The iron was measured using external calibration with multi-element standards at the wavelength of 238.2 nm. The accuracy of the analysis was checked by analyzing the NIST standard reference materials Typical Diet (SRM 1548a). Iron content was expressed as % w/w.

### 2.3 Microanalysis

Elemental analysis C, H, N and S was performed at the Neotron SPA laboratories in Modena (Italy). Results were obtained as % of C, H, N, S and converted in molar amounts by dividing the percentage to the respective atomic mass.

### 2.4 Synthesis of ferrous picolinate

The synthesis of ferrous picolinate was adapted by the procedure from Evans (17). In short, ferrous sulfate (20 g, 0.07 mol) was dissolved in 200 mL of Milli-Q® water (Millipore Milli-Q® Academic A10 18 mΩ/4 ppb) using a 250 mL beaker. Thereafter, the picolinic acid (20 g, 0.16 mol) was added to the solution and stirred continuously. After 3–5 min, a red precipitate began to form. After 30 min, the stirring was stopped and the mixture was left at room temperature until the precipitate had settled to the bottom of the beaker. The supernatant was removed and the precipitate was recrystallized by dissolving it in 200 mL of boiling Milli-Q® water followed by a cooling in an ice bath. The sample was stored for 16 h at 4 °C. Afterwards, the supernatant was removed from the crystals and the resulting wet mass was freeze-dried. The formation of the complex was detected visually through the appearance of an intense red precipitate.

### 2.5 Iron fortification and thermal processing of shelf-stable milky desserts with fruit

Fortification of shelf-stable milky desserts with fruit was prepared by mixing 0.8 mg of iron/100 g product, corresponding to 15% of the iron labelling reference value established for foods intended for young children. This level represents the minimum micronutrient addition required to ensure a meaningful contribution to the daily recommended iron intake, in accordance with European regulations (18). Subsequently, 35 g aliquots of the fortified samples were filled in 50 mL glass bottles (type Schott Duran) and flushed with argon gas for 30 s. Afterwards, the samples were autoclaved at 105 °C for 5 min. A non-fortified control sample was prepared in a similar manner as described above and used as a reference.

### 2.6 Color stability of fortified food

In order to quantify the changes in color before and after fortification, color analysis was carried out using a reflectance spectrometer (X-Rite ColorEye 7000A) set to a D65 illuminant and a 10° observer. Values of a* (the amount of red and green), b* (the amount of yellow and blue), L* (the amount of luminosity from black to white) were recorded in triplicate from three independent samples obtained for each treatment. The total color deviation (Δ*E*) of each sample was calculated according to Equation 1.

$$\Delta E=\sqrt{{\left(L_{\text{control}}^{*}-L_{\text{sample}}^{*}\right)}^{2}+{\left(a_{\text{control}}^{*}-a_{\text{sample}}^{*}\right)}^{2}+{\left(b_{\text{control}}^{*}-b_{\text{sample}}^{*}\right)}^{2}}$$ (1)

Δ*E* refers to a measure of the overall color change in the sample.

### 2.7 Shelf-life stability

Iron-fortified shelf-stable milky desserts with fruit with selected iron fortificants were prepared following the same procedure described earlier for fortification of shelf-stable milky desserts with fruit. Fortified and unfortified products were stored at 37 °C and monitored for color stability over a period of 4 months.

### 2.8 Iron bioaccessibility using human intestinal Caco-2 cells

*In vitro* digestions of fortified samples followed by ferritin measurement in Caco-2 cells was used as a method for measuring food iron availability (19). Fortified products underwent a two-step *in vitro* digestion: (1) gastric digestion with pepsin at pH = 2, 37 °C for 1 h; (2) intestinal digestion with pancreatin and bile at pH = 7, 37 °C for 2 h that took place on a dialysis membrane positioned directly on Caco-2 cell monolayers. After 18 h, ferritin measurements were used to quantify iron uptake by Caco-2 cells. Enzymes and buffers were used as negative control. Ferric chloride (FeCl_3_; 6 μg/mL) without food matrix was used as positive control.

Results were normalized by the total protein content and expressed as ng ferritin/mg protein ± SD (*n* = 9). The ferritin measurement was performed by enzyme immunoassay (Spectro Ferritin, Ramco Laboratories, Inc. TX, US). The protein content of the cells was measured using BCA protein assay (Pierce™ BCA Protein Assay Kit, Thermo Fischer Scientific, MA, US). The iron concentration was determined using the same method as reported above.

To ensure the robustness of results, experiments were carried out in triplicate on separate days using cells from the same batch with three replicates each day. Statistical comparisons to the reference compound (i.e., ferrous sulfate) was carried out with a paired *t*-test with two tailed distribution and two sample unequal variance.

## 3 Results and discussion

### 3.1 Chemical characterization

**Figure 1:**
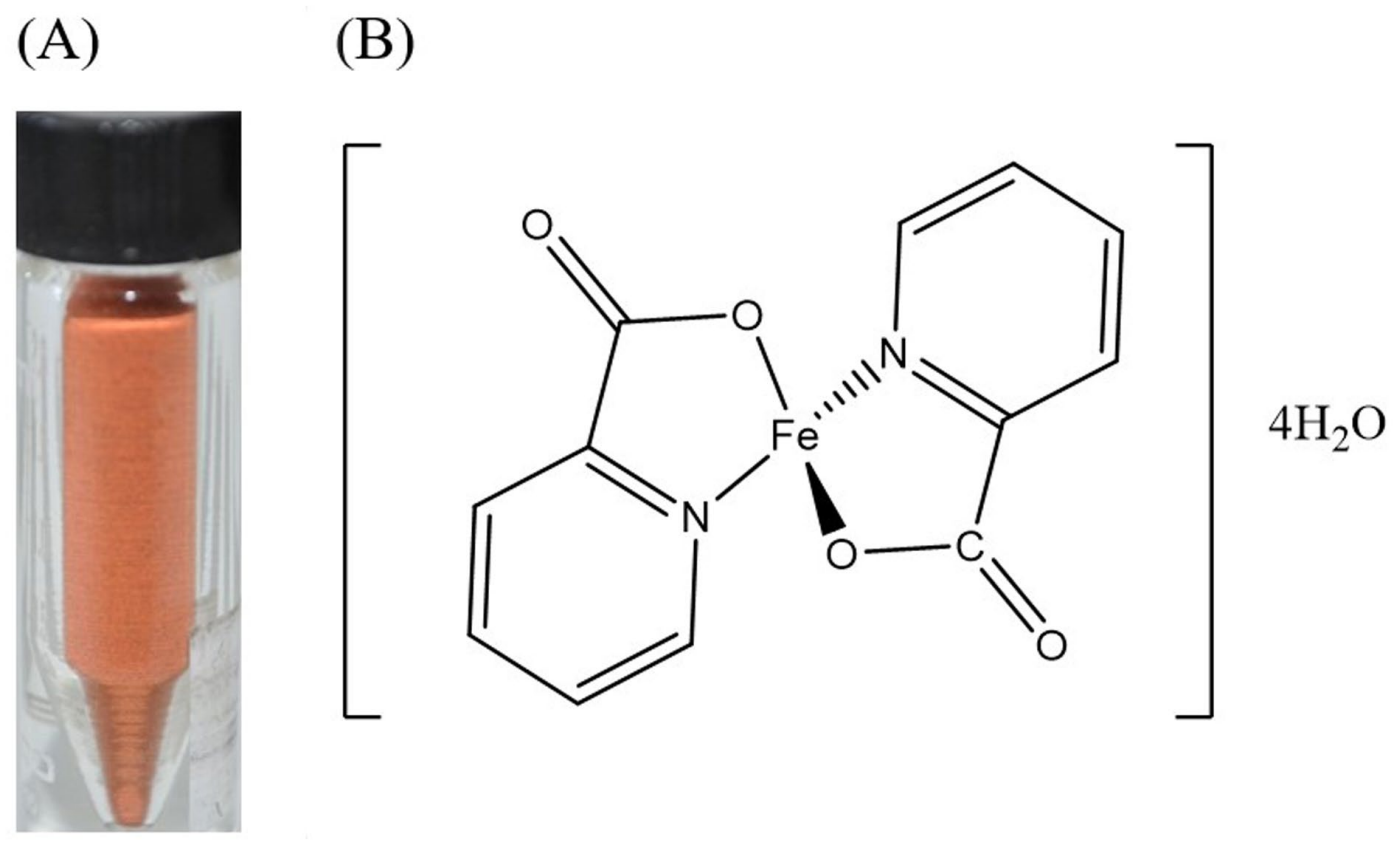
**(A)** Example of synthesized ferrous picolinate powder. **(B)** Chemical structures proposed for ferrous picolinate.

Picolinic acid, also known as pyridine-2-carboxylic acid, has a six-membered aromatic ring structure containing five carbon atoms, a nitrogen and a carboxylic group in position 2. Elemental and mineral analyses of the prepared sample yielded the following weight-by-weight (w/w) concentrations: %C 37.56, %H 4.28, %N 7.66, %S 0.32, and an iron content of 14.5%. Ferrous iron was prepared at 2:1 picolinic acid: ferrous sulfate molar ratio, which led to a red-orange chelated iron composition (Figure 1A) with the chemical formula C_12_H_16_O_8_N_2_Fe. The resulting powder and structure assigned to such formula calculated from the elemental analysis is shown in Figure 1. This structure is in agreement with previous reports (20).

Even though some authors reported the rapid oxidation of Fe^2+^ to Fe^3+^ in the complex at high pHs (21), the presence of only Fe^2+^ was confirmed through a combination of ferrozine test and ammonium thiocyanate (22, 23).

### 3.2 Color stability after heat treatment

**Figure 2:**
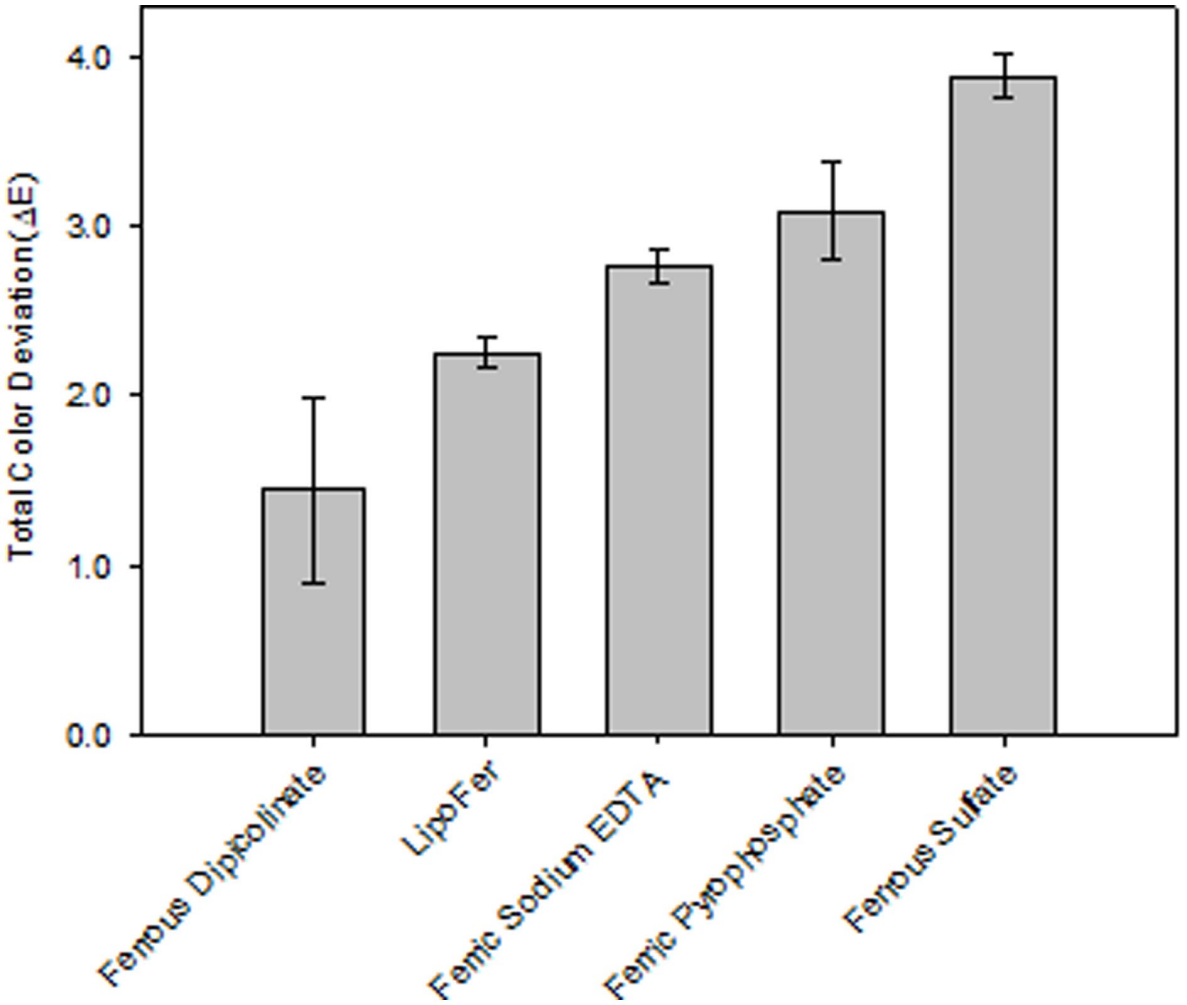
Effect of fortification on the color of heat treated (105 °C, 5 min) shelf-stable milky desserts with fruit.

The color stability of iron fortified shelf-stable milky dessert containing 25% fruit (15% banana; 10% strawberry) after heating with selected iron fortificants is shown in Figure 2.

Ferrous sulfate caused the most pronounced color changes due to its high solubility and reactivity with polyphenols, NaFeEDTA, and LipoFer™ showed improved stability but still led to noticeable color deviations. In contrast, ferrous picolinate maintained color stability, with Δ*E* values below 2 units, making fortified products indistinguishable from controls.

As expected, ferrous sulfate appeared to be the most detrimental fortificant given its high solubility in water, leaving iron readily available to interact with the polyphenols present in the product. Ferric pyrophosphate, the most recommended fortificant for matrices rich in phenolic compounds due to its low solubility in water (3), led to a lower color deviation but in general appeared to be less stable than NaFeEDTA and LipoFer™. In contrast, ferrous picolinate maintained color stability, with Δ*E* values below 2 units, making fortified products indistinguishable from controls.

Dairy products enriched with fruit serve as excellent vehicles for nutritional fortification, particularly for infants and toddlers. However, fruits present unique formulation challenges due to their high concentrations of native phenolic compounds (PC), including catechins, anthocyanins, and phenolic acids. These compounds act as effective coordination ligands for both ferrous and ferric iron, promoting interactions that can compromise product quality. While strategies such as the use of metal competitors or pH adjustments may delay the reaction between iron and gallol or pyrogallol groups in phenolic compounds (3), completely inhibiting this process remains difficult. The high mobility of iron in liquid or semi-liquid dairy matrices, combined with the acidic environment (pH ≈ 4.4), further facilitates iron’s reactivity with phenolic compounds.

As a result, iron–phenolic complexes typically impart a dark or grey coloration to the product, diminishing its luminosity and perceived freshness. In this study, the selected matrix—a milk-based dessert containing 15% banana and 10% strawberry—was estimated to contain approximately 40 mg gallic acid equivalents (GAE), corresponding to 0.23 mmol of total polyphenols derived from the fruit components (24) as well as 0.8 mg iron (0.014 mmol) per 100 g serving. The interaction between iron and phenolic compounds can be described by Equation 2:

$$\begin{array}{l} Fe^{\mathrm{II}}+n\mathrm{PC}⇌\mathrm{FeP}C_{n}\;\;(n=1-3)\\ \;\;\;\;\;\;\;\;\;\;\;\;\;\;\;\;\;\;K\beta_{n}=\frac{\left[\mathrm{FeP}C_{n}\right]}{[Fe^{\mathrm{II}}]\;{\left[\mathrm{PC}\right]}^{n}} \end{array}$$ (2)

Where [PC] is the concentration of phenolic compounds, [Fe^ıı^] is the concentration of iron, [FePC_*n*_] is the concentration of iron:PC complexes and K*β*_*n*_ is the stability constant of the complex.

Previously, Logβs has been reported to be in the range 7-9 for Fe^2+^: PC (25). Moreover, both iron and polyphenols must be available in solution to trigger the reaction.

Given the high stability constant and a substantial molar excess of phenolic compounds to iron (PC:Fe > 16), substituting the reported values into Equation 2 (with *n* = 3) and solving the resulting fourth-degree equation indicates that nearly all available iron is converted to Fe:PC complexes, exceeding 1.4 × 10^−2^ mmol. Consequently, the likelihood of forming dark-colored FePC_3_ complexes is extremely high under these conditions. This outcome aligns with previous observations by Habeych and colleagues, who reported similar complex formation when iron was combined with various fruit-derived phenolic compounds in matrices with pH values ranging from 3.6 to 5.6 (3). In parallel, Mansoor and Farooqui (26) reported that iron–picolinic acid complexes exhibit stability constants of a similar order of magnitude to those reported for iron–polyphenol complexes, while under the mildly acidic conditions typical of dairy matrices (pH 4–5), polyphenols remain predominantly in their protonated, phenolic form and display reduced iron-binding capacity, as described by Baldelli et al. (27). This pH-dependent ligand speciation supports a preferential interaction of iron with picolinic acid derivatives in such systems and underpins their suitability as iron fortificants in sensitive dairy products.

### 3.3 Shelf-life stability

**Figure 3:**
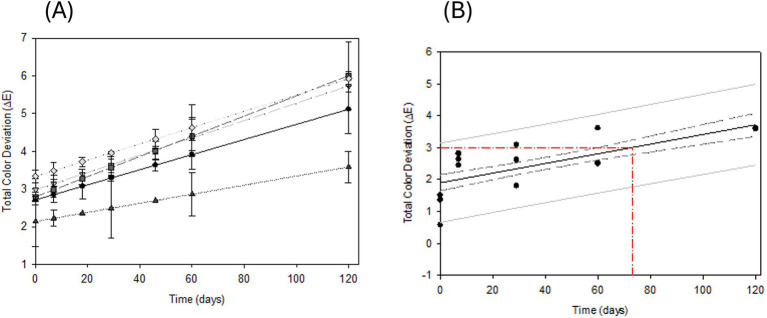
**(A)** Shelf-life stability of iron fortified shelf-stable milky desserts with fruit heat treated (105 °C, 5 min) and stored at 37 °C. (▲) Ferrous picolinate; (●) LipoFer™; (▼) ferric pyrophosphate; (■) ferric sodium EDTA; (**◇**) ferrous sulfate. **(B)** Prediction of onset off-color for ferrous picolinate fortified shelf-stable milky desserts with fruit.

Accelerated storage trials reveal that ferrous sulfate is the most detrimental among tested iron fortificants, causing the most significant color alterations in shelf-stable, iron-fortified milky desserts. In comparison, both ferric pyrophosphate (FePP) and sodium iron EDTA (NaFeEDTA) also contribute to noticeable discoloration, though to a lesser extent than ferrous sulfate (Figure 3A). The instability of NaFeEDTA might be due to zinc’s higher affinity for EDTA at pH 4.4 (28), displacing iron from the complex. Furthermore, heat treatment above 100 °C can oxidize EDTA, liberating Fe^3+^ ions that subsequently interact with polyphenols, exacerbating color instability (29).

Ferrous picolinate demonstrated robust color stability for over 120 days at 37 °C, effectively limiting the interaction between ferrous ions and polyphenols in the product. The observed stability cannot be solely attributed to the compound’s reported stability constants (logK*β*_*n*_ = 7.95 > > logK*β*_*n*_ = 5.4) (30), as its structure—comprising two picolinic acid ligands per Fe^2+^ ion—likely confers enhanced stability. Additionally, the presence of magnesium, calcium, and zinc may further inhibit polyphenol reactivity by occupying binding sites (3).

Accelerated shelf-life testing was conducted on milky desserts fortified with ferrous picolinate to assess the long-term color stability of the product. Regression analysis of the color stability data for different iron fortificants (Figure 3B) revealed that the degradation patterns of the various salts were statistically distinct (*p* < 0.01), indicating no significant correlation among their stability curves. The acceleration factor Q_17_ (k37/k20), representing the rate increase in color change when storage temperature rises from 20 to 37 °C, was previously determined to be 3.4.

Based on this modeling, the predicted shelf-life for a shelf-stable milky desserts with fruit fortified with ferrous picolinate exceeds 8 months at 20 °C before noticeable color changes occur. This duration aligns well with market requirements, as the standard shelf-life for such products is currently 9 months.

After 4 months of accelerated storage, ferrous picolinate demonstrated remarkable color stability in heat-treated shelf-stable milky desserts, remaining visually indistinguishable from the unfortified control. In contrast, other iron fortificants exhibited progressively greater color degradation over time, with changes proportional to the duration of storage. Notably, both NaFeEDTA and ferric pyrophosphate (FePP) showed substantial increases in Δ*E*ab* values throughout the shelf-life, mirroring the instability observed with ferrous sulfate.

### 3.4 Bioaccessibility of ferrous picolinate in Caco-2 cells

**Figure 4:**
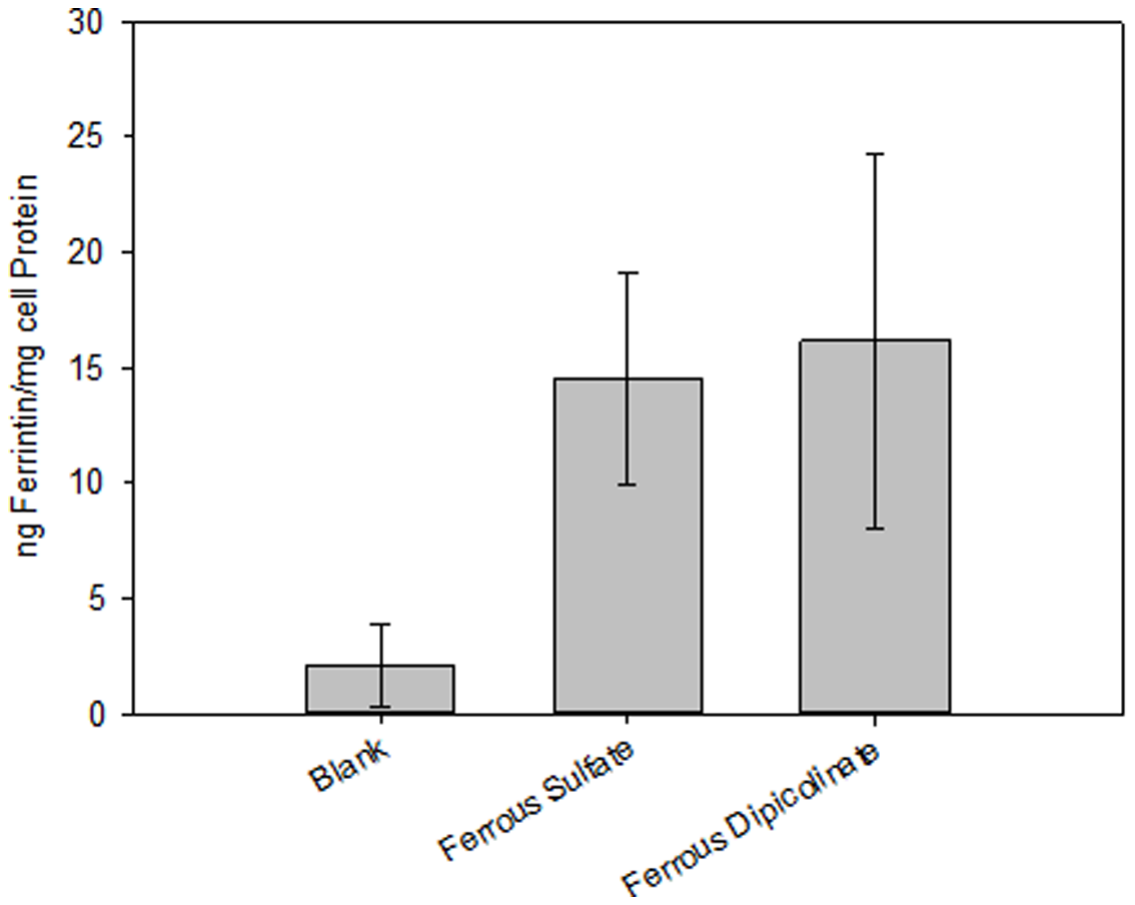
*In vitro* iron absorption from iron fortified shelf-stable milky dessert with fruit with selected iron fortificants via in-vitro digestion/Caco-2 cell culture assay. The final concentration was 100 μg iron/g product for all iron compounds. Values represent the mean + SD; *n* = 9.

Iron-fortified milky desserts containing 25% fruit significantly increased ferritin production in Caco-2 cells, indicating enhanced iron uptake, whereas the unfortified (control) dessert induced only minimal ferritin synthesis, comparable to baseline levels. The ferritin induction observed in the samples fortified with iron picolinate ranged from 16.2 ± 8.1 ng/mg protein as compared to a ferritin induction of 14.5 ± 4.6 ng/mg protein with the ferrous sulfate control (*p* = 0.59). Both types of fortified desserts exhibited significantly higher iron bioaccessibility compared to the unfortified control (*p* < 0.05). This ferritin induction from iron picolinate corresponds to 100% of bioaccessible iron compared to the ferrous sulfate control (Figure 4).

The levels of key inhibitors (e.g., phytic acid) and enhancers of iron absorption (e.g., vitamin C) were not quantified in the tested product, which limits interpretation of the present findings. Further studies are required to evaluate their potential influence on iron picolinate absorption, including investigations in other food matrices.

Importantly, the bioaccessibility of iron from ferrous picolinate was equivalent to that from ferrous sulfate, indicating that this novel compound can deliver iron as effectively as established fortificants. These *in vitro* results are further supported by *in vivo* study: a rigorously controlled human trial found that fractional absorption of iron from picolinate-fortified fruit yoghurt matched that of ferrous sulfate (16). Collectively, these results establish ferrous picolinate as an effective iron fortificant, matching the gold standard in bioavailability while offering additional formulation benefits, such as improved sensory stability.

## 4 Conclusion

Iron fortification in foods containing sensitive components, such as polyphenols, presents a dual challenge: maintaining desirable sensory properties while ensuring high iron bioavailability. Traditionally, these products are either left unfortified or use insoluble iron salts, which often result in modest nutritional outcomes.

This study demonstrates that ferrous picolinate is a promising new iron fortificant for polyphenol-rich dairy foods. It effectively preserves sensory qualities—such as color and taste—while delivering iron bioaccessibility equivalent to ferrous sulfate, the current gold standard. Its suitability for this matrix can be attributed to the combined effects of iron complex stability and the mildly acidic pH of the product, which together favor effective iron delivery without detrimental interactions with polyphenols.

Color stability, assessed via Δ*E*ab* analysis over 4 months of accelerated shelf-life, showed that ferrous picolinate maintains product appearance within a range acceptable to consumers. Predictive modeling suggests minimal visual changes for up to 8 months at ambient temperature, meeting market shelf-life requirements. Compared to reference iron salts such as NaFeEDTA and FePP, ferrous picolinate consistently outperformed in maintaining color stability.

Bioaccessibility studies using an *in vitro* Caco-2 cell model confirmed that iron from ferrous picolinate is as available for absorption as iron from ferrous sulfate, supporting its nutritional efficacy. The ready availability of picolinic acid also suggests that this approach could be cost-effective and scalable, pending regulatory approval.

In summary, ferrous picolinate offers a robust solution for iron fortification in sensitive food matrices, particularly those containing polyphenols. This strategy may be further extended by exploring other naturally occurring ligands, such as siderophores, to design stable and nutritionally valuable iron compounds for food applications.

## Data Availability

The datasets presented in this article are not readily available because all data presented in this manuscript are proprietary to Société des Produits Nestlé S. A. Requests to access the datasets should be directed to Edwin.Habeych@rd.nestle.com.
